# Beautiful Token

**Published:** 1888-03

**Authors:** 


### BEAUTIFUL TOKEN—WM. R. WARNER & CO.

The celebrated drug house of Wm. R. Warner & Co., Philadelphia, sent to the Editor of the RECORD one of their unique and beautiful parvule drug cases to be presented as a prize to the student passing the best examination in physiology. It was accordingly presented at the College Commencement, on the night of the 29th ult., to Dr. A. R. Stephens, who was the fortunate party securing the prize.

The parvules of Wm. R. Warner & Co. are gotten up with great care and neatness, only the purest drugs being used. The parvules are so graded that any size dose may be given, even to small children, and they have been found uniformly efficient in action.

---

THE February *Magazine of American History* is a model of elegance, and its contents are appetizing and delightful. The first article on the "Notable Editors between 1776 and 1800" affords twenty seven unique illustrations, including several of the rarest portraits known to picture collectors. Its author, Hon. S. G. W. Benjamin, illumines his text with quaint anecdote and felicitous quotation, and, as the result of much scholarly research, he furnishes fresh information (on a variety of points) which serves to demonstrate the marvelous influence of the early American press in the shaping of our public affairs. This series of papers touches a vital chord in the life of the country, and, as the field is vast and the harvesters few, its success is assured. Nothing now running as a serial through any periodical is calculated to attract more distinguished attention, or become more permanently valuable; the two chapters already published—in the January and February issue—are fully worth the yearly price of the magazine.

